# Frequency of difficult-to-manage and treatment-refractory axial SpA: insights from the German RABBIT-SpA register using recent ASAS definitions

**DOI:** 10.1093/rheumatology/keaf641

**Published:** 2025-12-03

**Authors:** Fabian Proft, Stephanie Lembke, Anja Weiß, Herbert Kellner, Xenofon Baraliakos, Denis Poddubnyy, Anne C Regierer

**Affiliations:** Department of Gastroenterology, Infectiology and Rheumatology (including Nutrition Medicine), Charité – Universitätsmedizin Berlin, Corporate Member of Freie Universität Berlin and Humboldt-Universität zu Berlin, Berlin, Germany; German Rheumatology Research Center (DRFZ Berlin), Epidemiology and Health Services Research, Berlin, Germany; German Rheumatology Research Center (DRFZ Berlin), Epidemiology and Health Services Research, Berlin, Germany; Private Practice for Rheumatology und Gastroenterology, Munich, Germany; Ruhr Universität, Bochum, Germany; Rheumazentrum Ruhrgebiet, Herne, Germany; Department of Gastroenterology, Infectiology and Rheumatology (including Nutrition Medicine), Charité – Universitätsmedizin Berlin, Corporate Member of Freie Universität Berlin and Humboldt-Universität zu Berlin, Berlin, Germany; Division of Rheumatology, University of Toronto and Schroeder Arthritis Institute, University Health Network, Toronto, Canada; German Rheumatology Research Center (DRFZ Berlin), Epidemiology and Health Services Research, Berlin, Germany

**Keywords:** axial spondyloarthritis, difficult-to-manage axSpA, treatment-refractory axSpA, treatment failure, real-world data, observational study, biologic therapy, patient-reported outcomes

## Abstract

**Objectives:**

To determine the frequency of axial SpA (axSpA) patients fulfilling the recently proposed Assessment of SpondyloArthritis International Society (ASAS) definitions for difficult-to-manage (D2M) and treatment-refractory (TR) axSpA, and to characterize these patients at initiation of their first advanced therapy.

**Methods:**

Data were derived from the ongoing prospective, multicentre, longitudinal German RABBIT-SpA registry. Patients were eligible if they were biologic and targeted synthetic (b/ts) DMARD-naïve, had initiated a b/tsDMARD and had ≥12 months of follow-up. ASAS definitions were applied to identify cases of D2M and TR.

**Results:**

Of 1850 patients with axSpA, 881 (48%) were b/tsDMARD-naïve at the start of observation. A total of 75/881 patients (8.5%) fulfilled the ASAS criteria for D2M and 22/881 (2.5%) additionally met the criteria for TR. At baseline, D2M patients were more frequently female, more often HLA-B27-negative, and more commonly presented with arthritis and enthesitis compared with not-D2M (nD2M) patients. In addition, they exhibited fewer objective inflammatory markers such as elevated CRP or MRI lesions. Opioid use was higher across D2M patients compared with nD2M patients. In the TR group compared with the D2M/nTR, the proportion of female and of obesity was lower. Even at initiation of first-line b/tsDMARD, these patients showed more frequently signs of inflammation, including sacroiliac/spinal MRI lesions and elevated CRP, while peripheral arthritis was less frequent.

**Conclusion:**

Applying the ASAS definitions in a large real-world cohort identified clinically relevant subgroups with D2M and TR. These findings support their clinical utility and highlight the need for phenotype-specific management strategies.

Rheumatology key messages

- Applying the ASAS consensus-based difficult-to-manage and treatment-refractory (D2M/TR) definitions in a real-world setting identified: 8.5% fulfilled D2M and 2.5% TR criteria.
- D2M patients were more frequently female, HLA-B27-negative, and more commonly presented with arthritis and enthesitis compared with nD2M-patients.
- D2M and TR patients displayed distinct clinical and inflammatory profiles, highlighting the need for phenotype-tailored management strategies.

## Introduction

Axial SpA (axSpA) is a chronic immune-mediated inflammatory disease primarily affecting the musculoskeletal system, particularly the sacroiliac joints and the spine [1, 2]. It is characterized by inflammatory back pain and stiffness, often leading to functional impairment and reduced quality of life in those affected.

As a heterogeneous condition with varying clinical manifestations, axSpA requires timely diagnosis and a multidisciplinary treatment approach aimed at controlling disease activity, alleviating symptoms and preventing long-term structural damage. In recent decades, substantial progress has been made in understanding the axSpA spectrum, including advances in disease classification, imaging and targeted therapeutic strategies [3]. Although significant advances have been made in the therapeutic management of axSpA [4], a considerable proportion of patients still experience suboptimal treatment responses. Data from randomized controlled trials with biologic and targeted synthetic DMARDs (b/tsDMARDs) indicate that only 40–50% of patients achieve a clinically meaningful treatment response, while remission or inactive disease states are reached by just 10–20% within 16–24 weeks of treatment initiation [5]. Potential contributors to these findings include disease-intrinsic mechanisms, as well as factors unrelated to active inflammation—such as non-nociceptive pain pathways, mechanical factors and comorbidities. Additionally, misclassification or misdiagnosis may play a role in the observed lack of treatment response. These observations underscore the need to better understand the mechanisms underlying treatment failure in this complex and heterogeneous patient population [6].

While the concept of difficult-to-treat disease is well established in RA [7, 8], no widely accepted criteria have been available for SpA. Recent efforts have begun to address this gap in both PsA [9, 10] and axSpA. To tackle the challenge of inadequate treatment response in axSpA, the Assessment of SpondyloArthritis International Society (ASAS) has proposed consensus-based criteria to identify two clinically relevant subgroups: difficult-to-manage (D2M) and treatment-refractory (TR) [11]. The D2M definition incorporates key elements such as treatment failure—defined as inadequate response to at least two b/tsDMARDs—persistent disease activity, and the shared perception of problematic signs and symptoms by both rheumatologists and patients. Within this framework, TR represents a more specific subset of patients who exhibit objective signs of active inflammation, such as elevated CRP levels or active inflammatory lesions on MRI in the sacroiliac joint (SIJ) or the spine, despite having received appropriate anti-inflammatory therapy. This group is distinct from patients whose persistent symptoms may be primarily driven by non-inflammatory mechanisms [11].

These definitions help identify patients who fail to respond adequately to standard therapies or continue to experience disease activity, posing unique clinical challenges. Understanding the characteristics of these subgroups is key to developing more individualized treatment approaches, reducing disease burden and improving long-term outcomes. Identifying the two subgroups, D2M and TR, represents a crucial first step toward understanding the diverse factors contributing to inadequate treatment response. The recently proposed ASAS definitions provide a standardized framework for identifying these challenging patient populations and facilitating targeted research into their underlying mechanisms, epidemiology and therapeutic needs.

The aims of this analysis were to prove the feasibility of the newly proposed D2M and TR criteria, to assess the frequency of axSpA patients meeting these criteria in a national, multicentre real-world setting [12], and to characterize these patients at the start of observation in the register.

## Methods

### Data source

RABBIT-SpA is a prospective, multicentre, longitudinal cohort study that has been ongoing in Germany since 2017. The study aims to evaluate the long-term safety and effectiveness of treatments involving b/tsDMARDs. Patients with a rheumatologist confirmed axSpA diagnosis are enrolled at the start of a new treatment [NSAIDs, conventional synthetic DMARDs (csDMARDs) or b/tsDMARDs] after prior pharmacological treatment failure (including NSAIDs) [12]. RABBIT-SpA is approved by the Ethics Committee of Charité University Medicine, Berlin (#EA1/246/16). All participants gave written informed consent. All patients are followed up at 3 months, 6 months and thereafter half-yearly up to a maximum of 10 years. At each follow up, in addition to clinical data obtained during the clinical appointments, patient-reported questionnaires are completed.

### Patient population

To qualify for inclusion in this analysis patients had to have axSpA, needed to be b/tsDMARD-naïve at the start of observation in RABBIT-SpA and had to have at least 12 months of follow-up observation time within the registry.

### Variables

Data were collected on sociodemographic and clinical characteristics as well as patient-reported outcome measures (PROMs). For this analysis, included sociodemographic characteristics factors were: sex (female/male), age (years), daily and occasional smoking (yes/no), educational level coded according to the International Standard Classification of Education (ISCED) [13] and BMI (kg/m^2^). Clinical and disease-specific factors encompassed: symptom duration (years), ASAS axSpA classification criteria fulfilled (yes/no), non-radiographic-axSpA (nr-axSpA, yes/no) [14], current inflammatory back pain (yes/no), Spondyloarthritis Research Consortium of Canada (SPARCC) Enthesitis Index (0–16 [15]), peripheral arthritis joint count (0–44 [16, 17]), active inflammation on MRI in the SIJ as well as spine (yes/no), CRP (<5/≥5 mg/l), HLA-B27 (positive/negative), extra-musculoskeletal manifestations (EMMs, ever occurred) including uveitis, psoriasis and IBD (yes/no), physician global assessment (PhGA) (0–10), Axial Spondyloarthritis Disease Activity Score (ASDAS [18]), modes of action (MoAs) of pharmacological treatment at start of observation, current use of NSAIDs, current csDMARDs, current opioid analgesics and current glucocorticoids (yes/no). Data regarding comorbidities (yes/no) were collected from the physician’s case report forms, where predefined conditions (e.g. cardiovascular disease, renal disease, malignancies, depression) are documented using a checklist, with the option to record additional conditions in free-text fields. Additionally, the Rheumatic Disease Comorbidity Index (RDCI, 0–9 [19, 20]) was calculated.

PROMs consisted of patient global assessment (PtGA, 0–10), pain (0–10), Assessment of Spondyloarthritis international Society Health Index (ASAS-HI, 0–17 [21–23]), BASDAI (0–10 [24]), fatigue measure via the BASDAI question 1 (0–10), BASFI (0–10 [25]) and the World Health Organization-Five mental well-being index (WHO-5, 0–100 categorized into validated cut-offs: moderate/severe depressive symptoms: 0–28, mild depressive symptoms: 29–50, well-being: >50 [26, 27]).

### Implementation of the ASAS definition criteria

**Figure 1.**
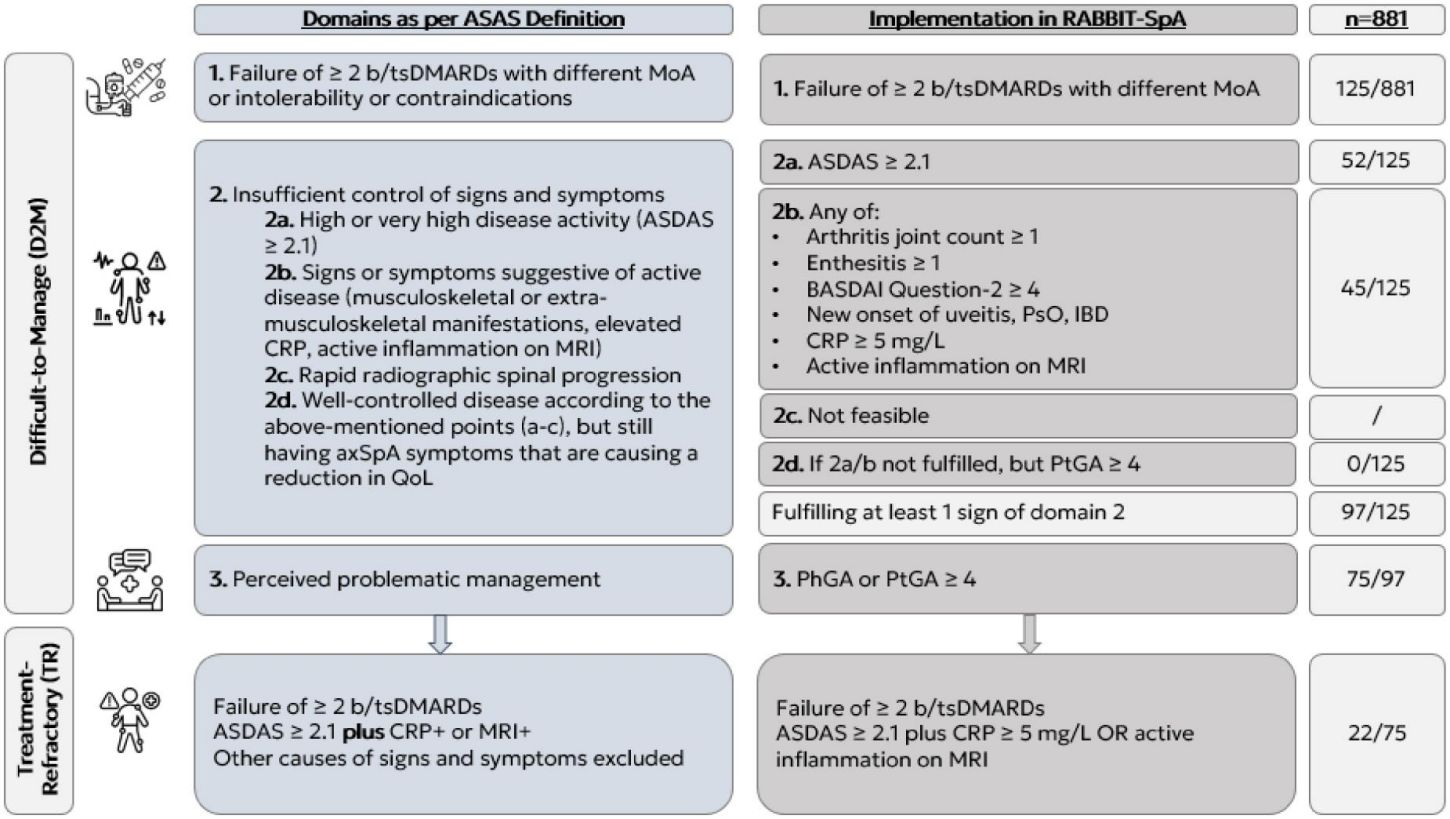
Feasibility and utilization of the ASAS D2M fefintion in RABBIT-SpA. In accordance with Poddubnyy *et al*. 2025 [11]

To identify D2M and TR patients the new ASAS criteria were applied [11]. The defined criteria were aligned to the variables available in our registry in order to guarantee their feasibility and applicability (Fig. 1).

To be included in the D2M group from the preselected cohort, patients need to fulfil the first criterion of the D2M definition: treatment failure of two or more b/tsDMARDs with different MoAs defined as treatment discontinuation because of an adverse event and/or lack of treatment efficacy (Fig. 1). The second D2M definition criterion, insufficient control of signs and symptoms, was assessed using the following indicators at the time point of the second b/tsDMARD failure: (2a) high disease activity: ASDAS ≥2.1; (2b) signs and symptoms suggestive of active disease, with at least one of the following present: arthritis joint count ≥1, enthesitis index ≥1, BASDAI question-2 (axial involvement) ≥4, new onset of uveitis, psoriasis or IBD, CRP ≥5 mg/l, active inflammation on MRI of the SIJ and/or spine; or (2d) PtGA ≥4. As sensitivity analysis, ASAS-HI ≥12/17 was utilized as the criterion for 2d. The criterion 2c (rapid radiographic spinal progression) is not adequately captured in the data set and was therefore omitted. The third D2M criterion was satisfied if either the PhGA or PtGA was ≥4 (Fig. 1).

Treatment refractory status was defined as patients meeting the D2M criteria and additionally a clear evidence of high disease activity (ASDAS ≥2.1) and either active inflammation on MRI of the SIJ/spine or an elevated CRP (≥5 mg/l) (Fig. 1).

### Statistical analysis

The longitudinal data were used to define whether the patient fulfils the D2M/TR definition. The patient characteristics are shown at the time point of inclusion into the register, that is, before the D2M/TR definitions had been met. The analysis focused on identifying the frequency of D2M and TR patients as well as to complete descriptive analysis of patient characteristics at the beginning of observation, for the following subgroups: not D2M (nD2M), D2M and TR. As no *a priori* hypothesis was formulated for statistical comparisons, conducting inferential statistical tests would not have been methodologically justified. Mean and s.d. were reported for continuous variables if normally distributed; otherwise, median and interquartile range (IQR) were provided. Categorical and binary variables were summarized using frequencies and percentages. No imputation of missing data was performed. All statistical analyses were conducted using SAS Enterprise Guide version 8.3. Database closure was 1 March 2024.

## Results

### Patient population

**Figure 2.**
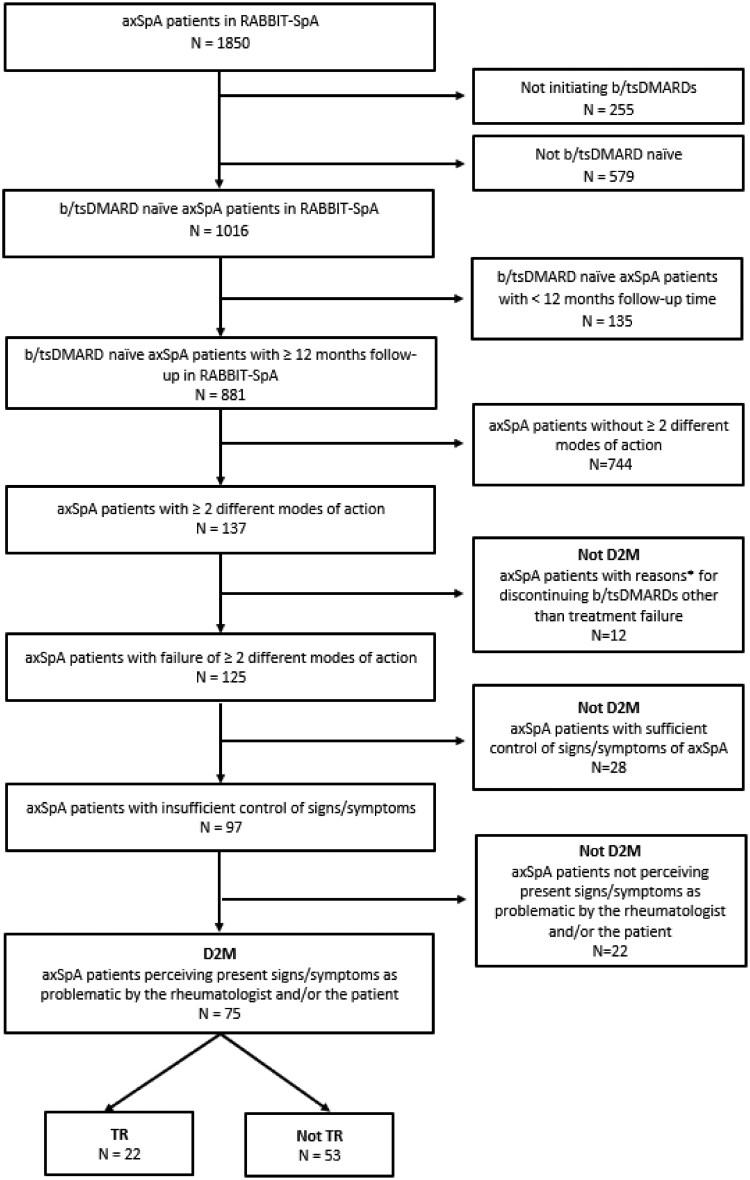
Flow chart of patient selection. ^a^Pregnancy, remission, family planning

A total of 1850 patients with axSpA from 86 recruiting centers were enrolled in the RABBIT-SpA registry. Of these, 1016 patients were b/tsDMARD-naïve at the time of inclusion (Fig. 2). Among them, 881 patients had a sufficient follow-up duration of at least 12 months and were therefore eligible for the analysis. At the start of observation the cohort comprised 43% female patients, with a mean age of 42.6 years (Table 1). The mean symptom duration was 11 years, and HLA-B27 positivity was observed in 76% of individuals. A total of 80% met the ASAS axSpA classification criteria, with 31% classified as nr-axSpA. The mean BMI was 26.6 kg/m^2^, and 40% of the participants were smokers. EMMs—uveitis, psoriasis and IBD—ever occurred in 14%, 9% and 5% of patients, respectively. Patient-reported outcomes indicated a mean BASDAI score of 4.7 and a mean BASFI score of 3.7, and 28% reported moderate to severe depressive symptoms. The mean follow-up time of the total cohort was 49 months.

**Table 1.** Patient characteristics of b/tsDMARDs-naïve axSpA patients with at least 12 months follow up time in RABBIT-SpA per non-D2M and D2M subgroup at the beginning of observation

		Missings, *n* (%)	nD2M, *n* = 806	D2M, *n* = 75	Total, *n* = 881
Sociodemographic factors					
Sex (female)	*n* (%)	0 (0)	339 (42)	40 (53)	379 (43)
Age (years)	Mean (s.d.)	0 (0)	42.4 (13)	45 (12)	42.6 (12.9)
Smoking (yes)	*n* (%)	97 (11)	282 (39)	28 (42)	310 (40)
ISCED 3	*n* (%)	94 (11)	209 (29)	9 (14)	218 (28)
BMI (kg/m^2^)	Mean (s.d.)	22 (2)	26.5 (5)	27.3 (5.4)	26.6 (5.1)
Obesity (BMI ≥30 kg/m^2^)	*n* (%)	22 (2)	166 (21)	20 (27)	186 (22)
Clinical factors					
Symptom duration (years)	Median (IQR)	5 (1)	6.8 (12.3)	7.8 (13.5)	6.9 (12.4)
Symptom duration (≤2 years)	*n* (%)	5 (1)	161 (20)	10 (13)	171 (20)
Diagnostic delay (years)	Median (IQR)	71 (8)	2 (5.5)	2.4 (9.7)	2 (5.5)
ASAS criteria fulfilled	*n* (%)	0 (0)	648 (80)	53 (71)	701 (80)
nr-axSpA	*n* (%)	479 (54)	116 (32)	9 (27)	125 (31)
HLA-B27 (positive)	*n* (%)	21 (2)	608 (77)	49 (68)	657 (76)
Current inflammatory back pain	*n* (%)	1 (0)	683 (85)	64 (85)	747 (85)
Enthesitis (current)	*n* (%)	5 (1)	138 (17)	20 (27)	158 (18)
Arthritis (current)	*n* (%)	4 (0)	219 (27)	25 (33)	244 (28)
Arthritis (≥5 joints)	*n* (%)	4 (0)	52 (7)	9 (12)	61 (7)
Active inflammation MRI SIJ (yes)	*n* (%)	231 (26)	482 (80)	40 (80)	522 (80)
Active inflammation MRI spine (yes)	*n* (%)	398 (45)	252 (58)	24 (51)	276 (57)
CRP (positive, ≥5 mg/l)	*n* (%)	57 (6)	434 (58)	36 (50)	470 (57)
Uveitis (ever)	*n* (%)	0 (0)	118 (15)	5 (7)	123 (14)
Psoriasis (ever)	*n* (%)	0 (0)	70 (9)	8 (11)	78 (9)
IBD (ever)	*n* (%)	0 (0)	39 (5)	2 (3)	41 (5)
Comorbidities (≥3)	*n* (%)	0 (0)	115 (14)	12 (16)	127 (14)
RDCI (0–9)	Mean (s.d.)	0 (0)	0.4 (0.8)	0.5 (0.9)	0.4 (0.8)
PhGA (NRS 0–10)	Mean (s.d.)	25 (3)	5.7 (1.7)	6.3 (1.6)	5.8 (1.7)
ASDAS	Mean (s.d.)	135 (15)	2.9 (1)	2.9 (0.6)	2.9 (1)
Treatments					
MoA at the start of observation	*n* (%)	0 (0)			
TNFi			638 (79)	45 (60)	683 (78)
IL-17i			110 (14)	18 (24)	128 (14)
JAKi			6 (1)	2 (3)	8 (1)
Controls			52 (6)	10 (13)	62 (7)
NSAIDs (current)	*n* (%)	1 (0)	576 (72)	48 (64)	624 (71)
csDMARDs (current)	*n* (%)	1 (0)	92 (11)	8 (11)	100 (11)
Opioids (current)	*n* (%)	7 (2)	54 (16)	8 (36)	62 (18)
Glucocorticoids (current)	*n* (%)	5 (1)	117 (15)	8 (11)	125 (14)
Patient-reported factors					
PtGA (NRS 0–10)	Mean (s.d.)	79 (9)	5.8 (2.4)	6.8 (1.7)	5.9 (2.4)
Pain (NRS 0–10)	Mean (s.d.)	80 (9)	5.6 (2.4)	6.7 (1.7)	5.7 (2.3)
ASAS-HI (0–17)	Mean (s.d.)	99 (11)	6.6 (3.4)	7.8 (3.4)	6.7 (3.4)
BASDAI (NRS 0–10)	Mean (s.d.)	79 (9)	4.6 (1.9)	5.3 (1.8)	4.7 (1.9)
Fatigue (NRS 0–10)	Mean (s.d.)	79 (9)	5.5 (2.4)	6.1 (2.6)	5.5 (2.4)
BASFI (NRS 0–10)	Mean (s.d.)	79 (9)	3.7 (2.3)	4.3 (2.2)	3.7 (2.3)
WHO-5 (moderate/severe)	*n* (%)	28 (3)	194 (27)	24 (36)	218 (28)

ASAS: Assessment of SpondyloArthritis international Society; ASAS-HI: Assessment of SpondyloArthritis international Society Health Index; ASDAS: Ankylosing Spondylitis Disease Activity Score; csDMARDs: conventional synthetic DMARDs; D2M: difficult-to-manage; HLA-B27: human leucocyte antigen; IL-17i: IL-17 inhibitor; ISCED: International Standard Classification of Education; JAKi: Janus Kinase inhibitor; MoAs: modes of action; nD2M: not difficult-to-manage; nr-axSpA: non-radiographic axial SpA; PhGA: physician global assessment; PtGA: patient global assessment; RDCI: Rheumatic Disease Comorbidity Index; SIJ: sacroiliac joint; TNFi: TNF inhibitor; WHO-5: World Health Organization-Five mental well-being index.

### Fulfilment of the D2M and TR definitions

During the observation period, among the selected 881 patients, 125 (14%) fulfilled the first criterion of the ASAS definition [failure (including primary/secondary failure, discontinuation because of side effects/intolerability/contraindications) of at least two b/tsDMARDs with different MoAs] (Figs 1 and 2). The second criterion according to the ASAS definition (insufficient control of signs/symptoms of axSpA) was met by 97 patients (Figs 1 and 2). Criterion 2a, ‘high or very high disease activity according to an ASDAS of ≥2.1’, was met by 52 patients, and criterion 2b, ‘signs or symptoms suggestive of active disease (musculoskeletal or EMMs, elevated CRP or active inflammation on MRI)’, by 45 patients. The criterion 2d, ‘well controlled disease according to the above-mentioned points, but still having axSpA symptoms that are causing a reduction in quality of life’, did not apply to any patient (Fig. 1). As sensitivity analysis, ASAS-HI ≥12/17 was utilized as the criterion for 2d (not shown), which did not result in any alteration in the frequencies of individuals fulfilling the D2M criteria compared with those who did not. Of the 97 patients who met the first and second criteria, a total of 75 patients met the third criteria. Thus, 8.5% of the 881 selected patients fulfilled the ASAS criteria for D2M (Figs 1 and 2).

Of the D2M patients, 22 patients (29% of 75) also showed persistent disease activity with an ASDAS of ≥2.1 and objective signs of inflammatory activity by either elevated CRP values (*n* = 17) or a positive MRI (*n* = 5) at the time point of second b/tsDMARD failure. The frequency of patients meeting the TR criteria was therefore 2.5% (22 of the 881 selected patients) (Figs 1 and 2).

### Comparison D2M to nD2M

When comparing the 75 patients in the D2M group with the 806 patients not meeting the D2M criteria (nD2M) at the beginning of observation in the register, the D2M included a higher proportion of females (53% *vs* 42%) and an older mean age (45 *vs* 42.4 years) (Table 1). A higher proportion of patients in the nD2M group had attained an educational level corresponding to ISCED 3, compared with those in the D2M group (29% *vs* 14%). The mean BMI was slightly higher across D2M patients (27.3 kg/m^2^), following a higher frequency of obesity (27% *vs* 21%). Clinically, symptom duration (median 7.8 years, IQR 13.5) was similar to the nD2M (median 6.8, IQR 12.3), but fewer D2M patients had an early disease course (13% *vs* 20%). The diagnostic delay was longer in D2M (6.2 *vs* 4.8 years). ASAS axSpA criteria fulfilment was lower in D2M patients than in nD2M patients (71% *vs* 80%). Enthesitis (27%) and arthritis (33%) were more frequent than in nD2M (17% and 27%, respectively). CRP positivity (50% *vs* 58%) and HLA-B27 positivity (68% *vs* 77%) was lower compared with nD2M. Comorbidities were slightly more frequent in the D2M group, particularly arterial hypertension (25% *vs* 19%) and depression (7% *vs* 5%) (see also Supplementary Table S1 available at *Rheumatology* online). Disease activity measured via the ASDAS was similar in both groups (mean 2.9 *vs* 2.9). However, disease activity measured via the PhGA was higher in the D2M group than in the nD2M group (mean D2M 6.3 *vs* mean nD2M 5.7). Use of NSAIDs was slightly lower across D2M patients (64% *vs* 72%), whereas current opioid use was higher across D2M patients (36%) compared with nD2M patients (16%). All PROMs indicated a higher disease burden in the D2M group (Table 1). The mean follow-up time in the nD2M group was 49 months and in the D2M group 52 months. The average time until second failure of the D2M patients was 35 months.

### Comparison of TR with D2M/nTR

When comparing the 22 patients in the TR group with the 53 patients D2M but not meeting the TR criteria (D2M/nTR), at the start of observation, the TR group was younger than the D2M/nTR group (43 *vs* 46 years) (Table 2). The proportion of females was lower (41% *vs* 59%). The frequency of obesity was lower in TR patients compared with D2M/nTR (19% *vs* 30%). The diagnostic delay was longer in TR (8.1 *vs* 5.3 years). HLA-B27 positivity was lower in TR compared with D2M/nTR (55% *vs* 74%). MRI evidence of inflammation was more pronounced in TR patients, especially in the spine (64% *vs* 46%). The majority of TR patients showed elevated CRP levels at the time of initiating their first b/tsDMARD therapy (73% compared with 40% in the D2M/nTR). Peripheral arthritis was less frequent in the TR compared with D2M/nTR. Most PROMs in the TR group were similar to those in the D2M/nTR group, except for BASFI, which was lower in the TR group compared with the D2M/nTR group (3.7 *vs* 4.5). The mean follow-up time in the nTR group was 51 months and in the TR group 55 months. The average time until second failure of the TR patients was 36 months.

**Table 2.** Patient characteristics per D2M/nTR and TR subgroup at the beginning of observation

		D2M/nTR, *n* = 53	TR, *n* = 22
Sociodemographic factors			
Sex (female)	*n* (%)	31 (59)	9 (41)
Age	Mean (s.d.)	46 (11.8)	42.7 (12.6)
Smoking (yes)	*n* (%)	20 (40)	8 (50)
ISCED 3	*n* (%)	33 (67)	14 (82)
BMI	Mean (s.d.)	27.3 (5.6)	27.3 (4.9)
Obesity (BMI ≥30)	*n* (%)	16 (30)	4 (19)
Clinical factors			
Symptom duration (years)	Median (IQR)	6.1 (13.9)	8 (8.5)
Symptom duration (≤2 years)	*n* (%)	8 (15)	2 (9)
Diagnostic delay (years)	Median (s.d.)	1.9 (6.6)	4.9 (9.7)
ASAS criteria fulfilled	*n* (%)	38 (72)	15 (68)
nr-axSpA	*n* (%)	6 (22)	3 (43)
HLA-B27 (positive)	*n* (%)	37 (74)	12 (55)
Current inflammatory back pain	*n* (%)	45 (85)	19 (86)
Enthesitis (current)	*n* (%)	14 (26)	6 (27)
Arthritis (current)	*n* (%)	20 (38)	5 (23)
Arthritis (≥5 joints)	*n* (%)	8 (15)	1 (5)
Active inflammation MRI SIJ (yes)	*n* (%)	30 (79)	10 (83)
Active inflammation MRI spine (yes)	*n* (%)	15 (46)	9 (64)
CRP (positive, ≥5 mg/L)	*n* (%)	20 (40)	16 (73)
Uveitis (ever)	*n* (%)	4 (8)	1 (5)
Psoriasis (ever)	*n* (%)	5 (9)	3 (14)
IBD (ever)	*n* (%)	2 (4)	0 (0)
Comorbidities (≥3)	*n* (%)	9 (17)	3 (14)
RDCI (0–9)	Mean (s.d.)	0.6 (0.9)	0.4 (0.8)
PhGA (NRS 0–10)	Mean (s.d.)	6.3 (1.6)	6.2 (1.6)
ASDAS	Mean (s.d.)	2.8 (0.6)	3.1 (0.8)
Treatments			
NSAIDs (current)	*n* (%)	33 (62)	15 (68)
csDMARDs (current)	*n* (%)	6 (11)	2 (9)
Opioids (current)	*n* (%)	5 (36)	3 (38)
Glucocorticoids (current)	*n* (%)	4 (8)	4 (18)
Patient-reported factors			
PtGA (NRS 0–10)	Mean (s.d.)	6.9 (1.9)	6.8 (1.3)
Pain (NRS 0–10)	Mean (s.d.)	6.5 (1.7)	7.1 (1.6)
ASAS-HI (0–17)	Mean (s.d.)	7.7 (3.5)	8 (3.4)
BASDAI (NRS 0–10)	Mean (s.d.)	5.3 (1.8)	5.1 (2)
Fatigue (NRS 0–10)	Mean (s.d.)	5.9 (2.7)	6.5 (2.3)
BASFI (NRS 0–10)	Mean (s.d.)	4.5 (2.3)	3.7 (1.9)
WHO-5 (moderate/severe)	*n* (%)	15 (31)	9 (53)

ASAS: Assessment of SpondyloArthritis international Society; ASAS-HI: Assessment of SpondyloArthritis international Society Health Index; ASDAS: Ankylosing Spondylitis Disease Activity Score; csDMARDs: conventional synthetic DMARDs; D2M: difficult-to-manage; HLA-B27: human leucocyte antigen; ISCED: International Standard Classification of Education; MoAs: modes of action; nD2M: not difficult-to-manage; nr-axSpA: non-radiographic axial SpA; PhGA: physician global assessment; PtGA: patient global assessment; RDCI: Rheumatic Disease Comorbidity Index; SIJ: sacroiliac joint; WHO-5: World Health Organization-Five mental well-being index.

## Discussion

This analysis from the German prospective, multicentre, longitudinal RABBIT-SpA registry provides important real-world evidence on the frequency and characteristics of patients with D2M and TR according to the recently proposed ASAS definitions. Among 881 b/tsDMARD-naïve patients with axSpA and sufficient follow-up, 9% met the criteria for D2M, while 3% fulfilled the more stringent criteria for TR.

Patients classified as D2M showed different characteristics even at the start of their first b/tsDMARD, hence before they fulfil the D2M definition. They were more often female, had slightly higher age at treatment initiation, were more often obese and had slightly more comorbidities. They showed more signs of peripheral involvement and all PROMs were worse. As per definition, the TR subgroup was marked by high disease activity in combination with objective inflammatory markers (elevated CRP and/or active MRI lesions) even at the initiation of their first b/tsDMARD therapy. Notably, TR patients reported similarly high subjective disease burden as the broader D2M group. Mental health burden was particularly pronounced in this subgroup. However, only 22 patients classified as TR in our cohort, limiting the findings for this subgroup. The characteristics of TR need to be confirmed in future real world data analyses. An important finding of our analysis is the high percentage of opioid use in the D2M and TR group compared with the nD2M/nTR. This highlights the inadequate control of the disease activity and illustrates an unmet need in these patients.

Two important studies that examined the D2T phenomenon in axSpA prior to the dissemination of the ASAS D2M definition warrant discussion. First, Philippoteaux *et al.* (2024) [28] analysed axSpA patients from three clinics in Northern France using the EULAR definition originally developed for D2T RA. They defined D2T axSpA as having failed two or more b/tsDMARDs with different MoAs, and very D2T (vD2T) as having failed two or more b/tsDMARDs within <2 years of follow-up. Among 311 patients with axSpA, 88 (28%) fulfilled the D2T definition, and 12 (4%) met the criteria for vD2T. Based on this analysis, the authors proposed criteria including three key domains for defining D2T in axSpA. The first domain relates to treatment failure, the second domain addresses evidence of ongoing disease activity or progression, and the third domain refers to the perception that disease management is challenging, as judged by the rheumatologist and/or the patient.

Building on this work, Saygın Öğüt *et al.* (2024) [29] applied these suggested criteria in a Turkish cohort deriving from one clinic comprising 166 patients. In this cohort, 38 patients (23%) fulfilled the criteria by Philippoteaux *et al.* In our analysis, the frequencies for D2M and TR patients were lower than those of Philippoteaux and Saygın Öğüt. This is likely due to divergent definitions used, as well as regional differences in prescribing behaviour influenced by health policy restrictions and the availability of b/tsDMARD therapies. Both previous studies identified certain characteristics, clinical and patient-reported, as being more frequent among patients with D2M disease. These characteristics included higher baseline BASDAI scores, more frequent peripheral arthritis and more comorbidities. Our findings support these observations; however, due to methodological differences caution is required when interpreting and generalizing these results. It is noteworthy, that most of the criteria proposed by Philippoteaux *et al.* (2024) have been considered in the ASAS criteria. The main difference between their definition and the newly proposed ASAS definition is the time and NSAID aspect which is not included in the new ASAS definition.

Moreover, Smits *et al.* (2025) [30] recently implemented the ASAS definitions in a Dutch cohort derived from two clinics. The results of the cross-sectional study demonstrated that 10% of the 236 included patients fulfilled the D2M criteria, while 2% fulfilled the criteria for TR. These findings are similar to our findings; however, it should be noted that there are substantial methodological discrepancies. Firstly, our study population was clearly defined as b/tsDMARD-naïve patients at the time of recruitment into the registry and were then followed prospectively. Secondly, while we presented clinical data on b/tsDMARD-naïve patients at the time when they initiated their first bDMARD or tsDMARD therapy, it remains unclear what time point the clinical data presented by Smits *et al.* is representing. Thirdly, the application of the ASAS criteria by Smits *et al.* shows some differences compared with our approach. For example, for criterion 2d they utilized ASAS-HI ≥12/17 and SF-36 PCS ≤40/100; we applied PtGA ≥4. To align the definitions used in our analysis with the one of Smits *et al.* we used ASAS-HI ≥12/17 in a sensitivity analysis as criterion 2d (not shown). This did not result in any alteration in the frequencies of individuals fulfilling the D2M criteria compared with those who did not. As for the third criterion, ‘perceived problematic management’, Smits *et al.*’s definition required a PtGA and/or PhGA ≥5/10, while we used a slightly lower threshold of ≥4/10 for either PtGA or PhGA. Nevertheless, in the absence of validated and recommended cut-off values, these definitions can be considered to be rather arbitrary. The criterion 2c ‘rapid radiographic spinal progression’ could not be depicted for Smits *et al.* as well as for this study, which may indicate a limitation of the criteria with regard to observational data. This has also been discussed by Wendling *et al.* (2025) [31]. Taken together, these analyses demonstrate the reproducibility of the ASAS definitions across real-world cohorts.

A major strength of this study is the use of a large, prospective and well-monitored registry with detailed clinical and patient-reported data. The implementation of the ASAS definitions in a longitudinal setting allows for systematic classification and offers insights into their feasibility in routine clinical practice. Nonetheless, several limitations warrant consideration. First, RABBIT-SpA includes only patients starting a new systemic treatment after prior treatment failure. This might introduce a selection bias towards more severe cases. Therefore, the ‘true frequency’ might be somewhat lower, potentially limiting generalizability to early or untreated axSpA populations. However, the here reported frequency is consistent with the previously published result [30]. Second, operationalizing the ASAS definitions required interpretation (e.g. domain 2d: well controlled disease, but still having axSpA symptoms that are causing a reduction in quality of life), and some elements (e.g. domain 2c: radiographic progression) could not be implemented. As a result, comparison across cohorts may be influenced by variable selection. Third, no inferential statistical analyses were performed. This decision was intentional, as the primary objective was to assess the feasibility of the proposed criteria and to describe the frequency and baseline characteristics of patients fulfilling the D2M and TR definitions. Since no *a priori* hypothesis for statistical comparison was formulated, conducting inferential testing would not have been methodologically appropriate. In addition, potential confounding factors—yet to be analysed—could influence comparisons between patient groups, and the relatively small sample sizes within the D2M and TR groups would limit the validity and power of such analyses. Another limitation is a rather high number of missing values in the imaging data. Within the RABBIT-SpA register MRI imaging is not always available. This reflects the fact that in Germany MRI is not part of routine clinical management for every patient. Lastly, the observational design of our study limits causal interpretation of our results.

From a research and policy perspective, this study illustrates how the ASAS framework can be applied to identify high-need patients within structured databases. Several questions remain open: Are the observed D2M/TR rates consistent across countries, and health systems? Can early clinical, imaging or molecular markers predict progression to these difficult states? What are the most effective multidisciplinary interventions for patients with high subjective burden but limited objective inflammation? And in truly refractory cases with persistent inflammatory activity, can dual or sequential therapies offer a favourable risk–benefit profile? Finally, harmonizing the operationalization of the ASAS definitions across cohorts will be essential to enhance comparability and guide personalized treatment strategies in axSpA.

## Conclusion

In this large, prospective, real-world cohort of patients with axSpA, we found that 9% fulfilled the recently proposed ASAS definition for D2M and 3% met criteria for TR. Notably, patients with TR reported a similarly high subjective symptom burden to those with D2M, but—by definition—showed greater objective signs of inflammation, such as elevated CRP or MRI lesions. The ASAS definitions proved applicable for analysing data derived from routine rheumatology care. They have the potential to identify clinically meaningful axSpA phenotypes that pose distinct therapeutic challenges. Their systematic implementation in clinical practice and research may support more personalized care pathways and serve as a foundation for future studies targeting disease mechanisms and treatment strategies in high-need axSpA populations.

## Supplementary Material

keaf641_Supplementary_Data

## Data Availability

In the RABBIT-SpA study, patients’ consent does not include approval to share their data.
