# Supplementary material for: Frequency of difficult-to-manage and treatment-refractory axial SpA: insights from the German RABBIT-SpA register using recent ASAS definitions
Source: Rheumatology (Oxford). 2025 Dec 3;65(1):keaf641. doi: 10.1093/rheumatology/keaf641 (PMC12822491; doi:10.1093/rheumatology/keaf641)
Supplement: keaf641_Supplementary_Data [file keaf641_supplementary_data.docx]

Supplementary Data

Table S1: Comorbidity data of b/tsDMARDs naïve axSpA patients with at least 12 months follow up time in RABBIT-SpA per non D2M and D2M subgroup at the beginning of observation

|  |  | **nD2M**  **n=806** | **D2M**  **n=75** | **Total**  **n=881** |
| --- | --- | --- | --- | --- |
| ***Comorbidities*** |  |  |  |  |
| Arterial hypertension | n (%) | 151 (19) | 19 (25) | 170 (19) |
| Coronary heart disease | n (%) | 18 (2) | 1 (1) | 19 (2) |
| Heart failure | n (%) | 1 (0.1) | 0 | 1 (0.1) |
| Cardiac conduction disturbance | n (%) | 7 (1) | 0 | 7 (1) |
| Occlusive peripheral arterial disease | n (%) | 2 (0.2) | 0 | 2 (0.2) |
| Stroke | n (%) | 2 (0.2) | 0 | 2 (0.2) |
| Polyneuropathy | n (%) | 1 (0.1) | 1 (1) | 2 (0.2) |
| Diabetes Type I | n (%) | 2 (0.2) | 1 (1) | 3 (0.3) |
| Diabetes Type II | n (%) | 20 (3) | 2 (3) | 22 (3) |
| Chronic obstructive pulmonary disease | n (%) | 14 (2) | 2 (3) | 16 (2) |
| Bronchial asthma | n (%) | 24 (3) | 4 (5) | 28 (3) |
| Malignant neoplasia | n (%) | 12 (2) | 0 | 12 (1) |
| Chronic renal failure | n (%) | 12 (2) | 2 (3) | 14 (2) |
| Hyperlipoproteinaemia | n (%) | 30 (4) | 2 (3) | 32 (4) |
| Chronic viral hepatitis | n (%) | 5 (1) | 2 (3) | 7 (1) |
| Other chronic liver disease | n (%) | 14 (2) | 0 | 14 (2) |
| Peptic ulcer disease | n (%) | 4 (1) | 0 | 4 (1) |
| Latent tuberculosis | n (%) | 6 (1) | 1 (1) | 7 (1) |
| Hip osteoarthritis | n (%) | 22 (3) | 4 (5) | 26 (3) |
| Knee osteoarthritis | n (%) | 30 (4) | 2 (3) | 32 (4) |
| Degenerative spinal disease | n (%) | 112 (14) | 7 (9) | 119 (14) |
| Osteoporosis | n (%) | 16 (2) | 4 (5) | 20 (2) |
| Sjögrens syndrome | n (%) | 1 (0.1) | 0 | 1 (0.1) |
| Rheumatoid arthritis | n (%) | 8 (1) | 1 (1) | 9 (1) |
| Fibromyalgia | n (%) | 5 (1) | 1 (1) | 6 (1) |
| Migraine | n (%) | 22 (3) | 2 (3) | 24 (3) |
| Depression | n (%) | 36 (5) | 5 (7) | 41 (5) |
| Other psychological disorders | n (%) | 4 (1) | 1 (1) | 5 (1) |
| Note: Only comorbidities with available data are shown. | | | | |
